# Compound danshen dripping pills normalize a reprogrammed metabolism of myocardial ischemia rats to interpret its time-dependent efficacy in clinic trials: a metabolomic study

**DOI:** 10.1007/s11306-019-1577-3

**Published:** 2019-09-20

**Authors:** Nan Aa, Jia-Hua Guo, Bei Cao, Run-Bin Sun, Xiao-Hui Ma, Yang Chu, Shui-Ping Zhou, Ji-Ye Aa, Zhi-Jian Yang, He Sun, Guang-Ji Wang

**Affiliations:** 1 Department of Cardiology, The First Affiliated Hospital of Nanjing Medical University, 300 Guangzhou Avenue, Nanjing, 210029 China; 2 Jiangsu Province Key Laboratory of Drug Metabolism and Pharmacokinetics, State Key Laboratory of Natural Medicines, China Pharmaceutical University, 24 Tongjia Xiang, Nanjing, 210009 China; 3 Tasly R&D Institute, Tianjin Tasly Group Co., Ltd., Pujihe East Road, Tianjin, 300410 China; 4 Nanjing Drum Tower Hospital, The Affiliated Hospital of Nanjing University Medical School, Nanjing, 210009 China

**Keywords:** Metabolomics, Myocardial ischemia, Compound danshen dripping pills

## Abstract

**Introduction:**

Clinical trials of Compound danshen dripping pills (CDDP) indicated distinct improvement in patients with chronic stable angina. Daily fluctuation of therapeutic effect agreed with a peak-valley PK profile during a 4-week CDDP regimen, but stabilized after 8-week treatment.

**Objectives:**

This article aims to explore the underlying mechanism for the time-dependent drug efficacy of the up-down fluctuation or stabilization in clinic trials.

**Methods:**

A rat model of myocardial ischemia was established via isoproterenol induction. Metabolomics was employed to analyze the energy-related substances both in circulatory system and myocardium in the myocardial ischemia model.

**Results:**

CDDP treatment ameliorated myocardial ischemia, reversed the reprogramming of the metabolism induced by ISO and normalized the level of most myocardial substrates and the genes/enzymes associated with those metabolic changes. After 1- or 2-week treatment, CDDP regulated plasma and myocardial metabolome in an analogous, time-dependent way, and modulated metabolic patterns of ischemic rats that perfectly matched with the fluctuated or stabilized effects observed in clinical trials with 4 or 8-week treatment, respectively.

**Conclusion:**

Metabolic modulation by CDDP contributes to the fluctuated or stabilized therapeutic outcome, and is a potential therapeutic approach for myocardial ischemia diseases.

**Electronic supplementary material:**

The online version of this article (10.1007/s11306-019-1577-3) contains supplementary material, which is available to authorized users.

## Introduction

The heart is a unique organ that has a large and constant energy demand but has limited capacity for storing energy. An efficient, plentiful and continuous supply of energy is crucially important to guarantee that the heart can function normally and to ensure that blood is pumped throughout the body. Myocardial ischemia occurs when there is insufficient coronary flow, which impairs the delivery of myocardial substrates and oxygen and hence is a metabolic incidence that is usually accomplished by changes in the metabolism and decreases in the energy supply of cardiomyocytes and the body. Although metabolic properties of myocardial ischemia have received global attention, and metabolic modulation has been expected as a promising therapeutic approach for ischemic heart diseases (Lopaschuk et al. 2010; Stanley et al. 2005), few agent has been widely applied, except for trimetazidine (Kantor et al. 2000). Compound danshen dripping pills (CDDP, T89, FDA clinic trial, also known as Dantonic), is a typical traditional Chinese medicine (Fufang Danshen Diwan, in Chinese) composed of Radix Salvia miltiorrhiza, Radix Notoginseng and Borneolum. CDDP has been approved by the CFDA (China Food and Drug Administration) since 1993 and shows great potential for use in the management of myocardial ischemia. Clinical trials from the US FDA II examining CDDP showed significant improvements in exercise tolerance in patients with chronic stable angina pectoris. The therapeutic effects included an up-down or a daily peak-valley wave mode as the fluctuation of PK curves after 4 weeks of CDDP intervention (Supporting information, Fig. S1). However, the fluctuations flattened out after 8 weeks of CDDP administration. In other words, the PK-PD and therapeutic outcomes at 4 and 8 weeks of CDDP treatment were not consistent (Guo et al. 2016).

Although, plasma and urine metabolomics studies suggested that CDDP regulated glycolysis, fatty acids metabolism, amino acids metabolism and polyol metabolism either based on a model after ligation of the left ventricular coronary artery or induced by isoproterenol (ISO), the effects on myocardiocytes and ischemic heart tissue were not evaluated (Xin et al. 2013; Zou et al. 2015). In this study, we established a model of myocardial ischemia using consecutive injections of low doses of isoproterenol and applied the model to assess the effects of CDDP on metabolism. A GC/MS-based metabolomics platform was used to profile the endogenous molecules, energy substrates in the circulatory system and heart tissues (Aa et al. 2005). Metabolic patterns and pathways were investigated to evaluate the modulation effect of CDDP on myocardial ischemia in the model.

## Materials and methods

### Agents and materials

The capsule of Compound danshen dripping pills (T89, Dantonic) was provided by Tasly Pharmaceutical Co., Ltd. (Tianjin, China), 270 mg per capsule. The principal components in CDDP were assayed and quantitatively controlled according to the quality specification (Supporting information, Table S1). The stable-isotope-labeled internal standard compound and other agents, organic solvents, purified water were acquired as previously reported (Guo et al. 2016) (Supporting information, Method S1).

### Animals, experimental design and samples

A total of 150 healthy male Sprague–Dawley (SD) rats were housed in polypropylene cages, with free access to the diet and water. The animals were randomly divided into three groups: (1) Control group (Z); (2) ISO model group (ISO); and (3) CDDP treatment group (CDDP + ISO) (Fig. S2). The myocardial ischemia model rats were induced by ISO (15 mg/kg), and the CDDP treatment group was given with CDDP (167 mg/kg) after an initial induction of ISO for 3 days, Method S2. The ISO group and CDDP treatment group each contained two subgroups: a 1-week treatment group and a 2-week treatment group. At the end of the experiment, the rats were anesthetically sacrificed, the plasma, heart and liver tissues were collected, prepared and stored either at − 70 °C or placed in 10% formalin solution for the pathological analyses. All animals were handled according to the guidelines of the Tasly Animal Research Committee, and the experimental protocols were approved by the Animal Ethics Committee of Tasly Institute (TSL-IACUC-2013-015). All animal procedures conform to the NIH guidelines on the protection of animals.

### Biochemical assays and histological inspections

The ratio of the heart weight (HW) to the body weight (BW) was calculated for each rat. The histological inspection was performed after embedding in paraffin wax (Leica EG1150, Germany) and cut into 3–5 μm slices (Leica RM 2235, Germany). One transversal section from the base area of the left ventricle was rehydrated and stained with hematoxylin and eosin (H&E) and examined via light microscopy (Nikon 80i, Japan) at 100× magnification by an experienced observer who was blinded to the groups.

### Echocardiography

One week after treatment with CDDP, cardiac function and basic parameters of heart were detected with a 30 MHz central frequency scan head and a high-frequency ultrasound system, Vevo2100 (Visual Sonics Inc, Toronto). Rats were first anesthetized with 1–2% isoflurane vapor. Then two-dimensional echocardiographic views were taken in ventricular short axis.

In M-mode, the left ventricular end-systolic diameter (LVES.d), left ventricular end-diastolic diameter (LVED.d), left ventricular posterior wall end-systolic thickness (LVPWTs), left ventricular posterior wall end-diastolic thickness (LVPWT.d), interventricular septum end-systolic thickness (IVSs) and interventricular septum end-diastolic thickness (IVS.d) were measured. While left ventricular ejection fraction (LVEF) and left ventricular fractional shortening (LVFS) were calculated as reported (Wu et al. 2010).

### GC/MS analysis, identification and the acquirement of quantitative data for the endogenous molecules in the plasma and heart tissue

The plasma and heart tissue samples were pretreated, extracted, and derivatised in the same way as reported (Guo et al. 2016). The quality control (QC) samples were prepared from a pool of plasma or heart tissues, respectively, with the same procedure as the above samples. To minimize systematic variations, all samples were analysed at random order, with QC samples inserted. The raw GC/MS data were processed using Shimadzu GC postrun analysis software (Shimadzu GC/MS solution 2.0). Each of the endogenous compounds was identified and the quantitative data was acquired in the same way as reported (Guo et al. 2016). The system stability and analysis consistency were assessed by checking the QC samples, intensity of internal standard, myristic-1,2-^13^C_2_ acid, and an external reference standard, methyl myristate. The variation of all the samples was evaluated by the intensity of the internal standard, with the RSD less than 15%. The stability of the GC/MS system was evaluated by the intensity of the external standard, with the RSD less than 10%.

### Multivariate statistical analysis and pathways analyses

After acquirement of the data for each analyte, a data matrix can be constructed and normalized against the IS, with the sample names as the observations in the first column, peaks/analytes/molecules as the response variables in the first row, and the peak areas as the relative intensity. The constructed data matrix was then evaluated using SIMCA P14.0 software (Umetrics, Umeå, Sweden), (Bjerrum 2015; Trygg et al. 2007). Permutation test was assessed and PLS-DA model was calculated and validated to show the clustering of samples from different groups. Discriminatory compounds were extracted from loading plots constructed following the OPLS-DA analyses. Potential biomarkers, proteins and the genes within a metabolic pathway were identified based on databases such as KEGG (http://www.genome.jp/kegg/), HMDB (http://www.hmdb.ca/) and LipidMaps (http://www.lipidmaps.org/). The mRNA level of proteins/genes was assayed accordingly (Table S2, Method S3).

### Statistical analysis

In the present study, the data were expressed as the mean ± S.D. and were analyzed using one-way analysis of variance (ANOVA) tests in SPSS (version 18.0) with a significance level of 0.05 or 0.01.

## Results and discussion

### CDDP alleviated myocardial pathology induced by ISO

**Fig. 1:**
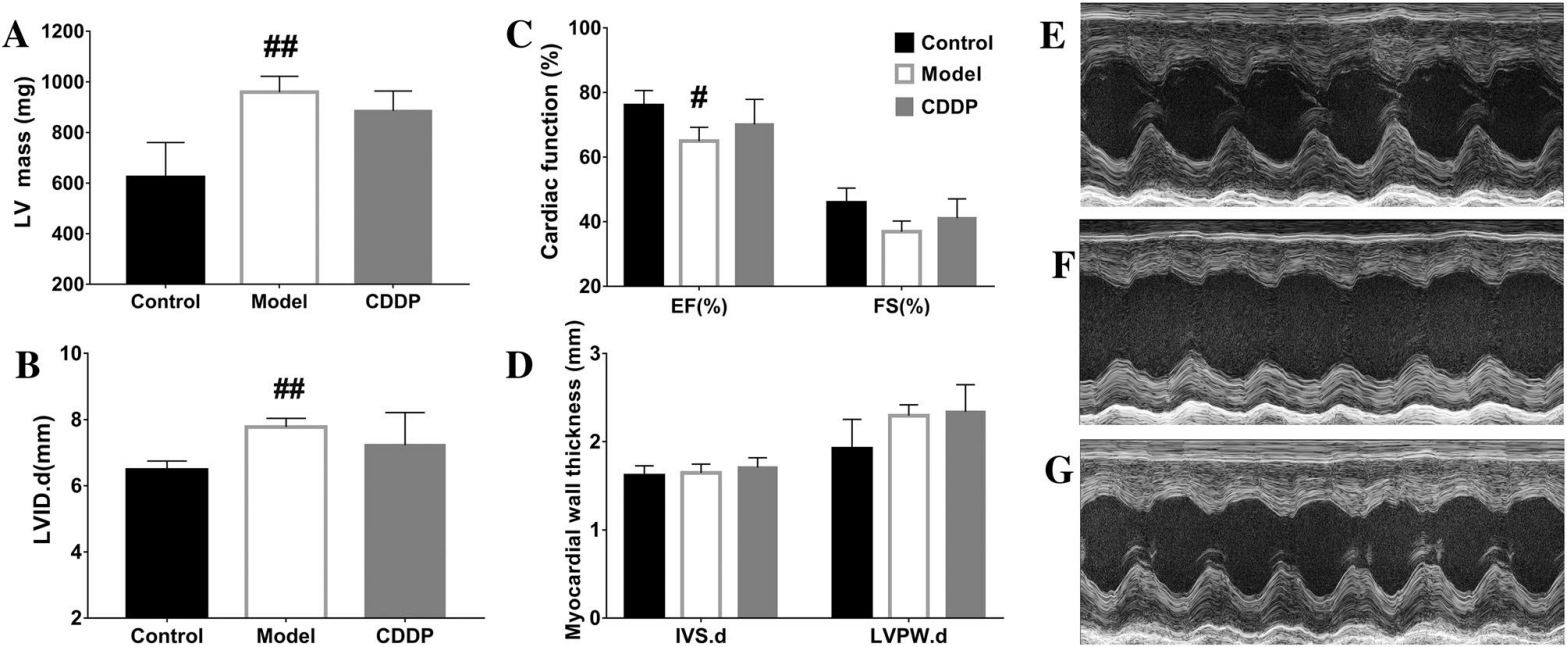
Cardiac functions and parameters of the rats assessed by echocardiographic examination. a Cardiac function assessed by ejection fraction (EF) and fractional shortening (FS); **b** thickness of interventricular septum (IVS) and left ventricular posterior wall (LVPW) in diastolic phase; **c** left ventricular internal diameter in diastolic phase; **d** left ventricular mass (corrected LV mass) calculated by echocardiographic parameters; **e–g** cardiac performance shown by typical echocardiographic snapshot of a rat from the vehicle control (n = 4), the ISO induced model (n = 3, one rat died during the anesthesia) and the CDDP treatment for 1 week (n = 4), respectively. **P < 0.01 vs vehicle control, P > 0.05 vs CDDP; *P < 0.05 vs vehicle control, P > 0.05 vs CDDP

Multiple injections of ISO increased the ratio of heart weight to body weight (HW/BW), attenuated cardiac remodeling of thicker myocardium, elevated LV mass and expanded left ventricle, induced myocardial injuries and necrosis, and reduced myocardial function of EF and FS. Treatment with CDDP alleviated the tendency induced by ISO, although without statistical significance for a few of the parameters, Fig. 1, Fig. S3, Table S3.

### Metabolic patterns of plasma fluctuated 2 and 24 h after CDDP treatment for 1 week, but stabilized after 2 weeks

**Fig. 2:**
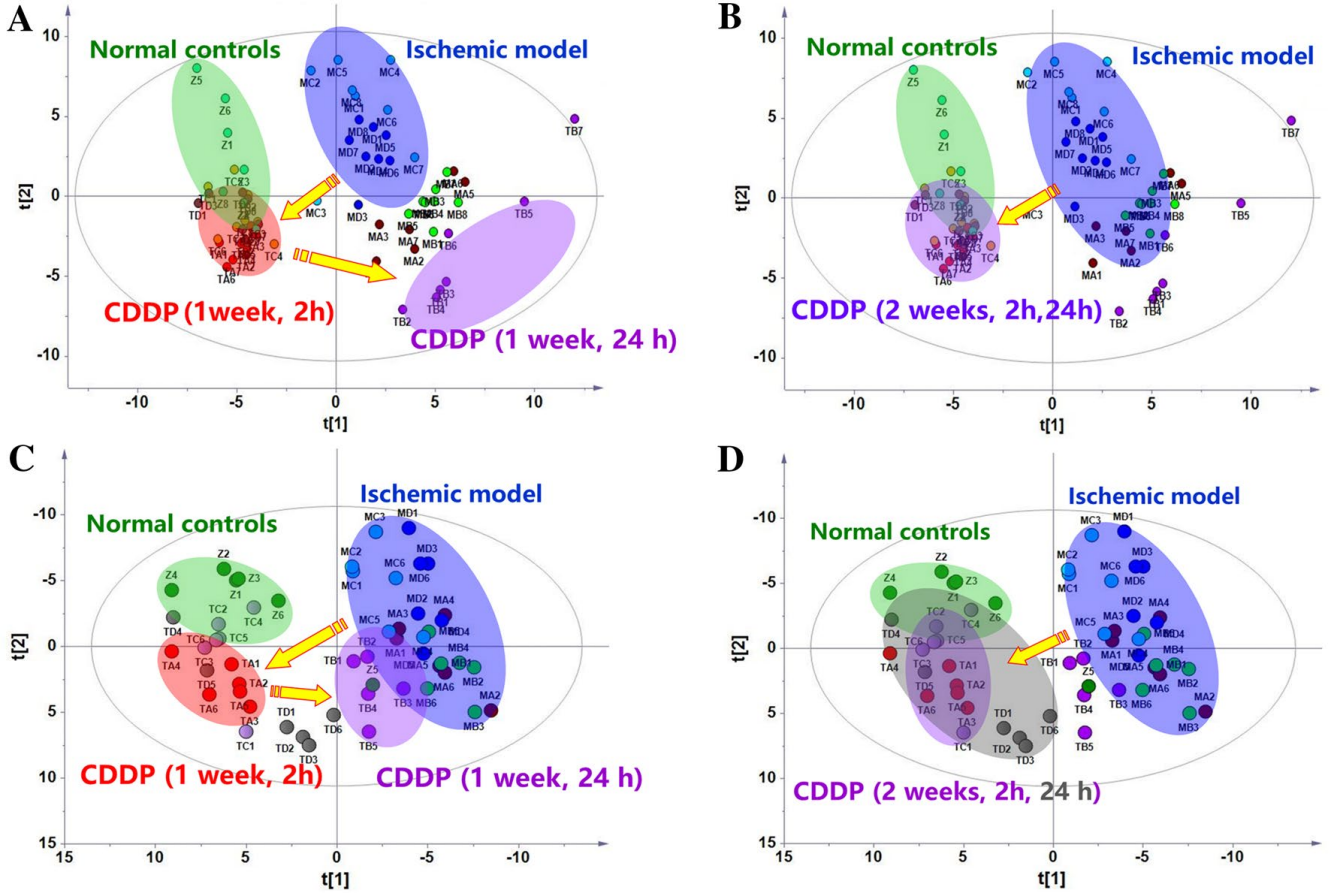
The reprogrammed metabolic patterns of the ischemia myocardial model rats and the changes in metabolic patterns by 1 or 2 weeks of CDDP treatment based on the PLS-DA score plots of the plasma and heart tissue metabolomes (n = 6). **a** plasma metabolome, 1 week; **b** plasma metabolome, 2 weeks; **c** heart metabolome, 1 week; **d** heart metabolome, 2 weeks. Parameters of the two models: **a** R^2^X = 0.597, R^2^Y = 0.849, Q^2^ = 0.701; **b**: R^2^X = 0.397, R^2^Y = 0.93, Q^2^ = 0.78; **c** R^2^X = 0.628, R^2^Y = 0.915, Q^2^ = 0.717; D: R^2^X = 0.613, R^2^Y = 0.916, Q^2^ = 0.714. Z: control; MA and MB: samples collected at 2 h and 24 h, respectively, in model rats induced by a 1-week injection of ISO; MC and MD: samples collected at 2 h and 24 h, respectively, in model rats induced by a 2-week injection of ISO; TA and TB: samples collected at 2 h and 24 h, respectively, in rats treated with CDDP for 1 week; TC and TD: samples collected at 2 h and 24 h, respectively, in rats treated with CDDP for 1 week

GC/MS analysis of the plasma samples aligned the analytes in the typical chromatograms and provided the quantitative data for each analyte in the plasma samples of the model, the control and the CDDP treatment groups, Fig. S4. Deconvolution of the chromatograms produced 120 peaks in the plasma extracts, where 73 analytes were authentically identified, Table S4. Based on the relative abundance of each peak in data matrix, a supervised PLS-DA model was created that designated the samples into nine groups: ISO-treated animals at 2 and 24 h of the two experimental stages (1 week and 2 weeks), CDDP-treated animals at 2 and 24 h of the two stages, and the normal control animals. The score plots of the samples tended to cluster closely within each group. Regardless of which samples were collected at 2 or 24 h, induction with ISO for either 1 week (2 h, MA; 24 h, MB) or 2 weeks (2 h, MC; 24 h, MD) caused a distinct deviation of the results from the samples from those of the normal controls (Z), Fig. 2a. Specifically, after 1 week of CDDP treatment, the samples from 2 h (TA) after CDDP administration were closer to the normal controls, indicating that the rat model’s metabolome was similar to that of the normal controls. This result indicates that CDDP markedly regulate the perturbed metabolism in the model animals at the peak of the PK profile, Fig. S1. However, the samples taken 24 h (TB) after CDDP administration were similar to the model controls, rather than the normal controls. It was suggested that CDDP did not regulate the perturbed metabolism in the model animals at the valley of the PK profile. This finding indicates that the modulation of the overall metabolism fluctuated with time, which is consistent with a peak-valley fluctuation of PK, Fig. S1.

However, unlike the results produced after 1-week treatment with CDDP, when CDDP was continuously administered for 2 weeks, the samples at both 2 h (TC) and 24 h (TD) overlapped with the normal controls and far from the ischemia rats, Fig. 2b. This finding indicates that the effects of CDDP on the metabolism stabilize over time, regardless of the peak-valley fluctuation of PK (data not shown). The similar regulatory effects on metabolic patterns in ischemic model rats 2 and 24 h after 2 weeks of treatment with CDDP agrees with clinical observations in patients who received CDDP for 8 weeks. It was indicated that the reversal of the metabolic disturbances was a result of the pharmacological effect of CDDP, and metabolic modulation represented therapeutic output in the clinic trials.

### Metabolic patterns of heart tissue fluctuated 2 and 24 h after CDDP treatment for 1 week, but stabilized after 2 weeks

GC/MS analysis profiled 181 peak molecules in the heart extracts, and 95 molecules were identified (Table S5). Similarly, A PLS-DA model was created that classified the tissue samples into the same nine groups used for the plasma samples (Fig. 2). As found in the plasma data, 1-week (2 h: MA; 24 h: MB) and 2-week (2 h: MC; 24 h: MD) ISO inductions both caused the samples to deviate significantly from the normal (Z), Fig. 2c, d, indicating strong metabolic disturbances of the molecules in the heart tissue. Treatment with CDDP reversed these deviations 2 and 24 h after 2 weeks of treatment (TC and TD) and 2 h after 1 week of treatment (TA), and all these samples clustered closely to the normal controls. Treatment with CDDP for longer time periods achieved a better metabolic modulation effect than treatment of shorter durations. The distinct modulation of metabolome in heart tissues suggested the modulation of CDDP on the perturbed metabolism of myocardiocytes induced by ISO. It was indicated that metabolic modulation of CDDP was associated with its therapeutic outcome observed in clinic trials. Considering that metabolic pattern of plasma and heart tissue represented the fundamental status of the whole body, a similar metabolic phenotype of CDDP treated group to that of the normal control suggested that CDDP comprehensively modulated the system metabolism of the model rats. The systematic modulation effect of CDDP on metabolism was similar and resembled to that of total ginsenosides, and agreeing well with holistic concepts of TCM in therapeutic mode (Shi et al. 2016; Aa et al. 2010b).

To quantitatively assess the regulatory effects, relative distance values (RDV) (Aa et al. 2010a) were calculated based on the data from the PLS-DA model, Fig. 2. The relative distance value M/N clearly showed a peak-valley fluctuation after treatment with CDDP on week 1 and a stabilized metabolic phenotype on week 2, and hence a week-dependent modulation on the perturbed metabolism in a quantitative way, Table 1.

**Table 1:** Assessment of the regulatory effect of CDDP on analytes/metabolism

Samples/period	The ratio of normalized analytes (%, A/B)		The ratio of relative distance values (M/N)	
	2 h	24 h	2 h	24 h
Plasma/1 week	84.4% (27/32)	34.2% (13/38)	0.43	1.36
Plasma/2 weeks	88.9% (24/27)	96.7% (29/30)	0.41	0.45
Heart/1 week	69.7% (23/33)	24.1% (7/29)	0.49	3.27
Heart/2 weeks	51.7% (15/29)	64.3% (9/14)	0.35	0.79

A, the number of analytes regulated by CDDP treatment; B, the number of discriminant analytes between the model and normal controls. M, the apparent distance values of drug treatment group to control group; N, the apparent distance values of drug treatment group to model group

### The ISO-induced alteration of energy metabolism and energy substrates in plasma and heart tissue was effectively regulated by CDDP

The heat maps showed the analytes altered by ISO induction and the regulatory effects of CDDP, Fig. S5. Both of the plasma and myocardial dendrograms showed marked changes in energy metabolism including fatty acids metabolism, glycolysis, TCA cycle and amino acids turnover in the rat models, although there were varied changes in plasma from those of heart tissue, Fig. S6. We also observed that the changes of analytes in plasma and heart tissue were efficiently reversed by CDDP (2 h and 24 h after 2 weeks of treatment and 2 h after 1 week of treatment), Tables S6–S7, Fig. S7. Totally, 84.4% (27/32) and 88.9% (24/27) discriminant analytes in plasma were significantly rectified 2 h after CDDP treatment for 1 and 2 weeks, respectively, Table 1; however, only 34.2% (13/38) and as high as 96.7% (29/30) discriminant analytes in plasma were regulated 24 h after CDDP treatment for 1 and 2 weeks, respectively. For the discriminant analytes in heart tissue, 69.7% (23/33) and 51.7% (15/29) discriminant analytes were significantly rectified 2 h after CDDP treatment for 1 and 2 weeks, respectively, Table 1; however, 24.1% (7/29) and 64.3% (9/14) discriminant analytes were regulated 24 h after CDDP treatment for 1 and 2 weeks, respectively. The percentage of analytes modulated by CDDP did not only suggest the consistent modulation on plasma analytes with those of tissue samples, but also showed the fluctuated or stabilized regulation in a week dependent way.

### Metabolic pathways and the key enzymes and transporters involved in the modulation of CDDP

The pathway analysis revealed that ISO markedly disturbed energy substrates and metabolic pathways including fatty acids, branch chain amino acids and glucose metabolism both in the blood and in the heart tissue, Fig. S6. Based on the modules of the metabolic networks, a phase diagram of a radar chart analysis were generated for the key energy substrates in glycolysis/TCA cycle and fatty acid metabolism. It clearly showed that CDDP treatment efficiently regulated glycolysis and TCA cycle homeostasis based on plasma data, except those at 24 h after 1 week of treatment, Fig. S8B; while CDDP markedly reversed fatty acids metabolism based on data of heart tissue, except those at 24 h after 1 week of treatment, Fig. S8C. Interestingly, the level of citrate and 3-hydroxybutyrate described the metabolic modulation of CDDP, suggesting the potential markers for both myocardial ischemia and the efficacy of CDDP treatment.

The mRNA levels of key enzymes and transporters analysis revealed that glucose transporters GLUT1, GLUT4 and HK-2 were significantly up-regulated in the cardiomyocytes of rats with myocardial ischemia. However, these enzymes returned to normal levels after 2 weeks of CDDP intervention, Fig. S9A. Similarly, ISO markedly promoted the expression of carnitine acyltransferases CPT1β and CPT2, the monocarboxylic acid transporters of MCT1 and MCT2, hepatic 3-hydroxy-3-methylglutaryl-CoA synthase 2 (HMGCS2) in the model rats, but the tendency was reversed by CDDP intervention, Fig. S9. *CD36* was down-regulated after ISO induction but was activated by CDDP intervention. On the other hand, however, 3-hydroxybutyrate dehydrogenase BDH2 was marginally up-regulated in rats treated with ISO, yet CDDP caused a tendency towards even higher levels, Fig. S9.

**Fig. 3:**
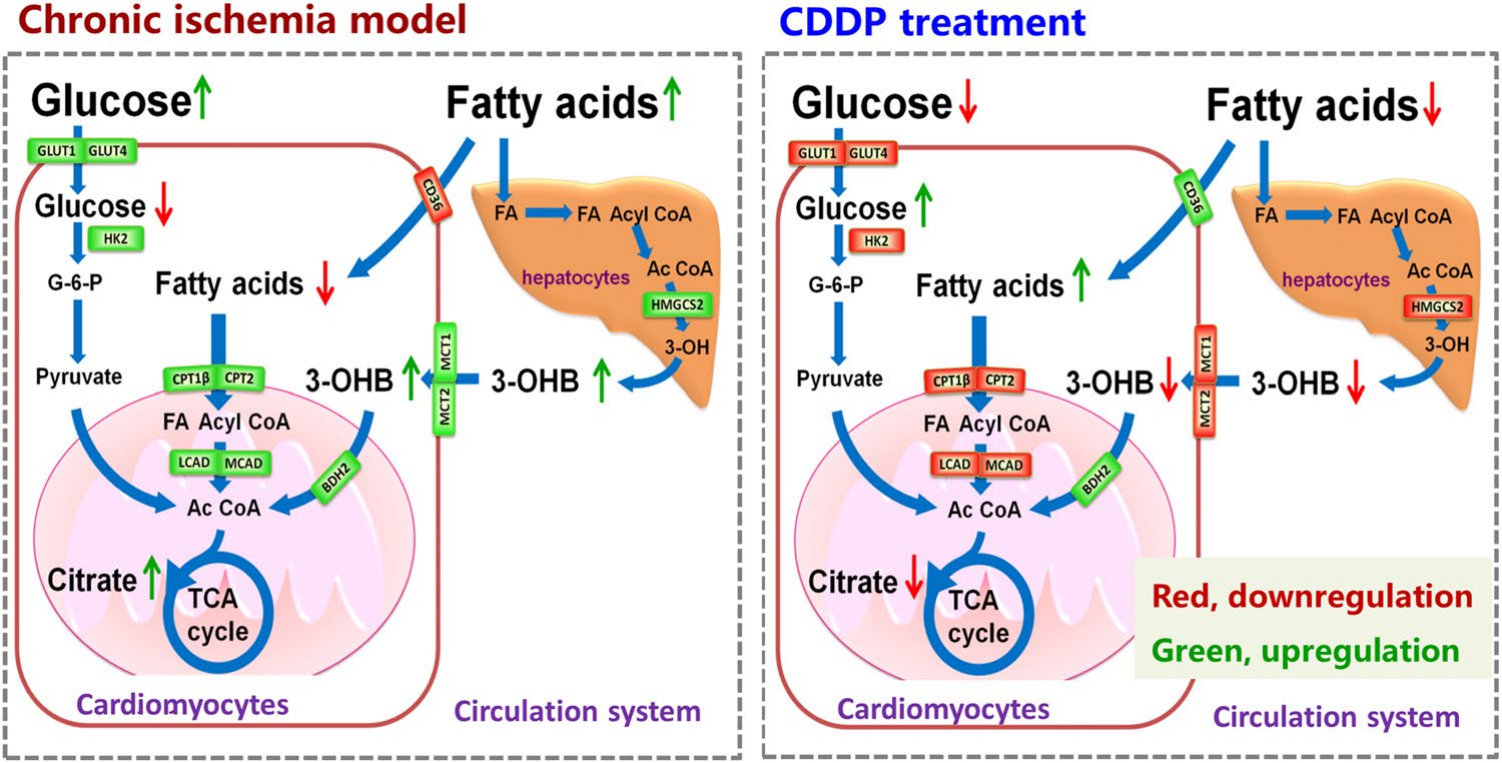
The key metabolism and energy substrates involved in ISO induction and treatment with CDDP in the myocardial ischemic rat model. CDDP gradually reversed the depression in fatty acid metabolism and enhanced glucose metabolism induced by ISO, based on metabolomics data and gene expression assay

In summary, in response to myocardial ischemia, cardiomyocytes tended to take up more glucose and ketone bodies instead of free fatty acids from the peripheral circulation system. In this condition, cardiomyocytes favored a metabolic reprogramming, which was shifted away from primarily relying on fatty acid metabolism to anaerobic glycolysis and the breakdown of ketone bodies, Fig. 3. In contrast, CDDP treatment reversed this metabolic reprogramming induced by ISO to a large extent. Due to the profound impact on metabolism in ischemic rats, the time-dependent response of the therapeutic outcome was predominantly dependent on changes in the metabolism rather than on the PK exposure of the pharmaceutical components in vivo. In other words, the modulation of energy metabolism could have been a primary cause of the pharmacological effects of CDDP on myocardial ischemia.

## Electronic supplementary material

Below is the link to the electronic supplementary material.

Supplementary Figures (Figs S1–S9) (DOCX 4131 kb)

Supplementary Tables (Tables S1–S7) (DOCX 61 kb)

Supplementary methods (Method S1–S3) (DOCX 20 kb)
