# Supplementary material for: Compound danshen dripping pills normalize a reprogrammed metabolism of myocardial ischemia rats to interpret its time-dependent efficacy in clinic trials: a metabolomic study
Source: Metabolomics. 2019 Sep 20;15(10):128. doi: 10.1007/s11306-019-1577-3 (PMC6754357; doi:10.1007/s11306-019-1577-3)
Supplement: Supplementary file 1 — Supplementary Figures (Figs S1–S9) (DOCX 4131 kb) [file 11306_2019_1577_MOESM1_ESM.docx]

**Compound danshen dripping pills normalizes a reprogrammed metabolism of myocardial ischemia rats to interpret its time-dependent efficacy in clinic trials: a metabolomic study**

**Supplementary Figures**


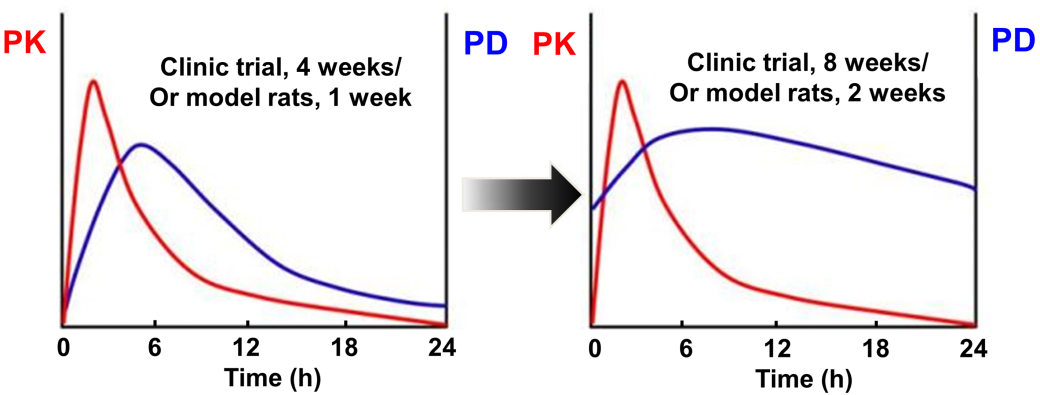


**Fig S1** Pharmacokinetics/pharmacodynamics (PK/PD) profiles and the alternation at different stages in the phase II clinical trial of Dantonic (CDDP, T89)


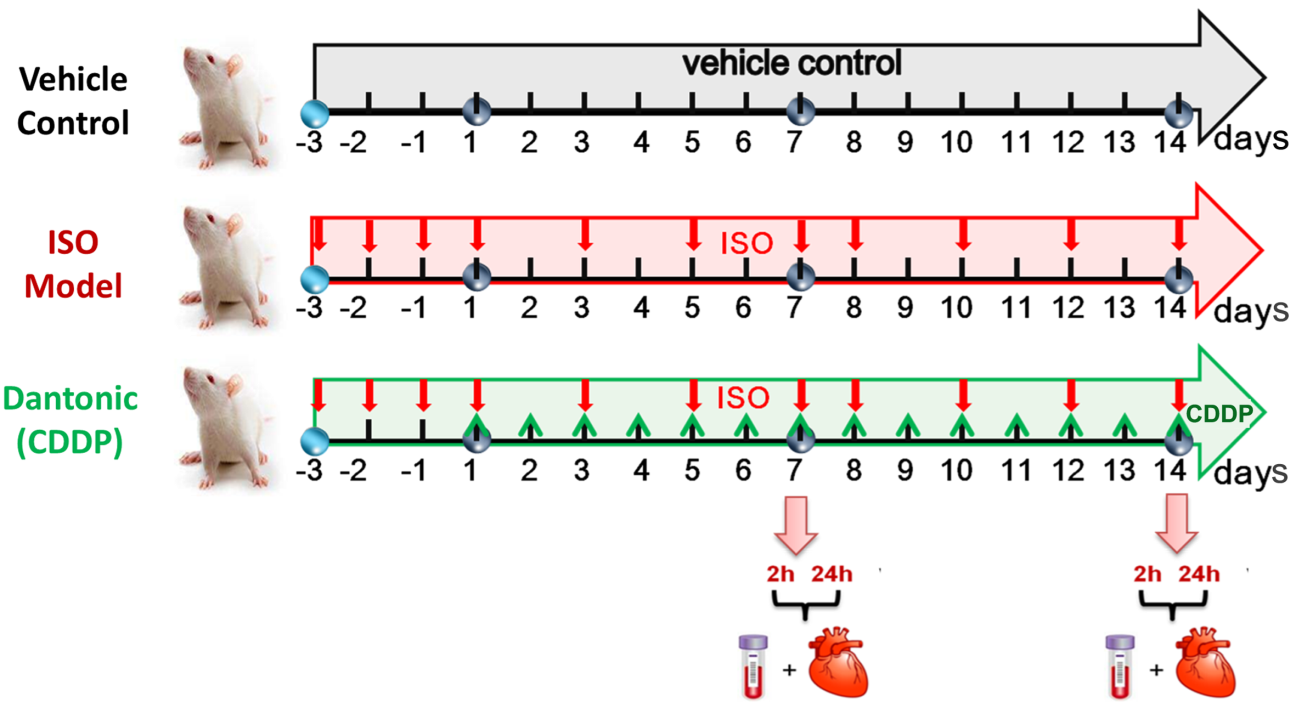


**Fig S2** A brief flow chart of the experimental design. ISO, isoproterenol; Dantonic, T89, FDA clinic trial, also known as Compound danshen dripping pills(CDDP)





**Fig S3** The HW/BW index and the pathological inspection of myocardial tissue of the ISO-induced model rats, the rats treated with CDDP and the normal control groups. A&B: heart weight and body weight index (HW/BW, n=6); C&F: staining in the normal control group (n=3); D&G: staining in the ISO model group(n=3); E & H: staining in the CDDP+ISO group(n=3). Data are presented as means ± S.D. (n =6). ***P*< 0.01 vs control; **P*< 0.05 vs control; ##*P* < 0.01 vs ISO model; #*P* < 0.05 vs ISO model.


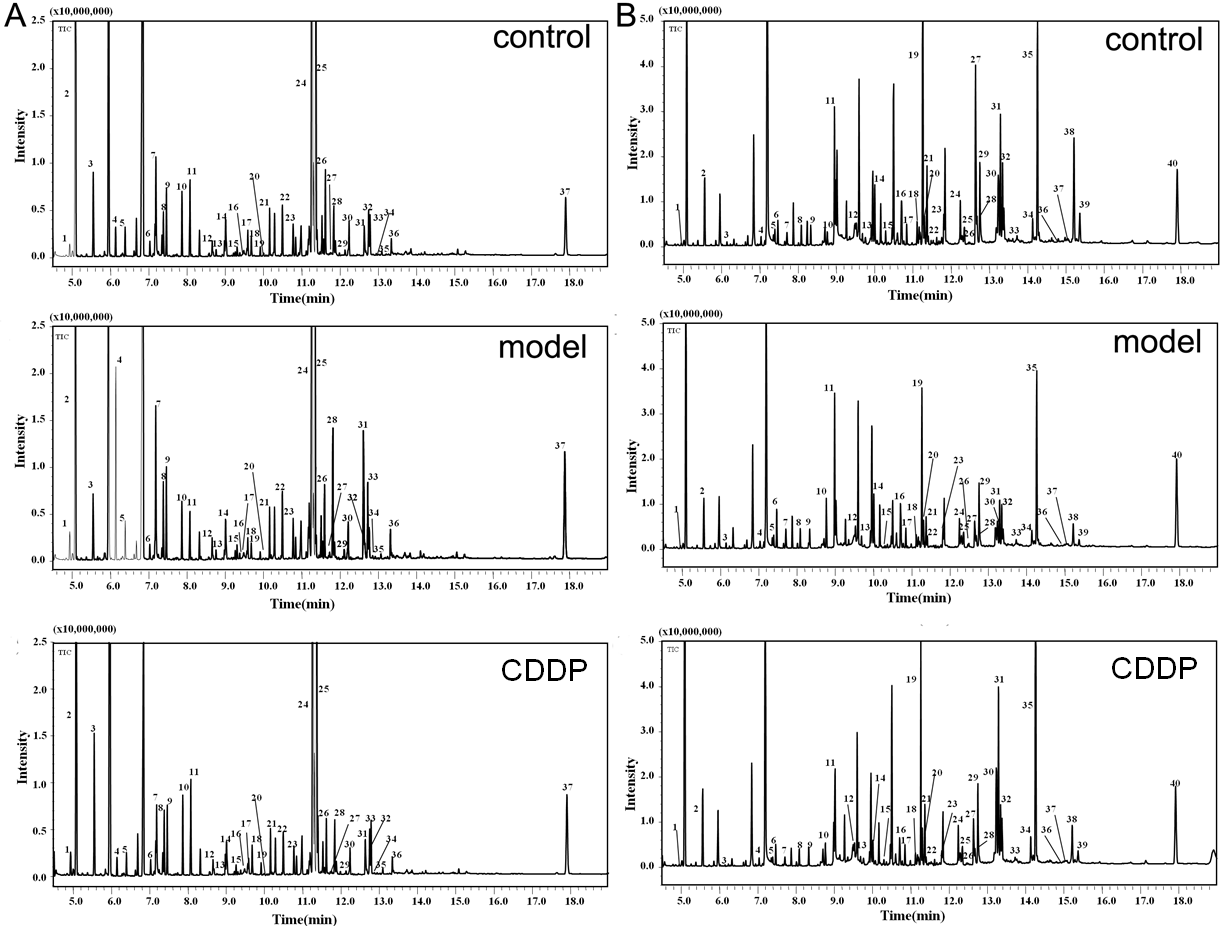


**Fig S4** Typical GC/MS chromatograms and the profiles of molecules in the plasma and heart tissue extracts for the ischemia model, Dantonic (CDDP, T89) treatment and the controls. A, plasma samples; B, heart tissue samples.

(A) Typical GC/MS chromatograms of plasma extracts. The molecules were identified as: 1:Pyruvate; 2:Lactate; 3:Alanine; 4:3-Hydroxybutyrate; 5:Urea; 6:Leucine; 7:Isoleucine; 8:Proline; 9:Glycine; 10:Serine; 11:Threonine; 12:Aspartate; 13:Malate; 14:5-Oxoproline; 15:Cysteine; 16:α-Ketoglutarate; 17:Ornithine; 18:Glutamate; 19:Phenylalanine; 20:Asparagine; 21:Lyxose; 22:Glutamine; 23:Citrate; 24:Glucose; 25:Tyrosine; 26:Palmitoleic acid; 27:Palmitoleic acid; 28:Palmitic acid; 29:Allantoin; 30:Heptadecanoic acid; 31:Linoleic acid; 32:Oleic acid; 33:Stearic acid; 34:Arachidonic acid; 35:Cystine; 36:Pseudo uridine; 37:Cholesterol;

(B) Typical GC/MS chromatograms of heart tissue extracts. The molecules were identified as: 1:Pyruvate; 2:Alanine; 3:3-Hydroxybutyrate; 4:Leucine; 5:Proline; 6:Glycine; 7:Fumarate,Uracil; 8:Threonine; 9:Aspartat; 10:Malate; 11:Aspartate; 12:Glutamate; 13:Phenylalanine; 14:Lyxose; 15:Glycero-3-phosphate; 16:Hypoxanthine; 17:Citrate; 18:Fructose; 19:Mannose; 20:Lysine; 21:Glucose; 22:Tyrosine; 23:Palmitoleic acid; 24:Ribulose 5-phosphate; 25:Ribose; 26:Glucose-6-Phosphate; 27:Linoleic acid; 28:Oleic acid; 29:Stearic acid; 30:Galactofuranose-6-Phosphate; 31:Mannose-6-Phosphate; 32:Arachidonic acid; 33:Uridine; 34:1-Monopalmitin; 35:Sedoheptulose-7-Phosphate; 36:1-Monolinolein; 37:1-Monostearin; 38:Raffinose; 39:Maltose; 40:Cholesterol.


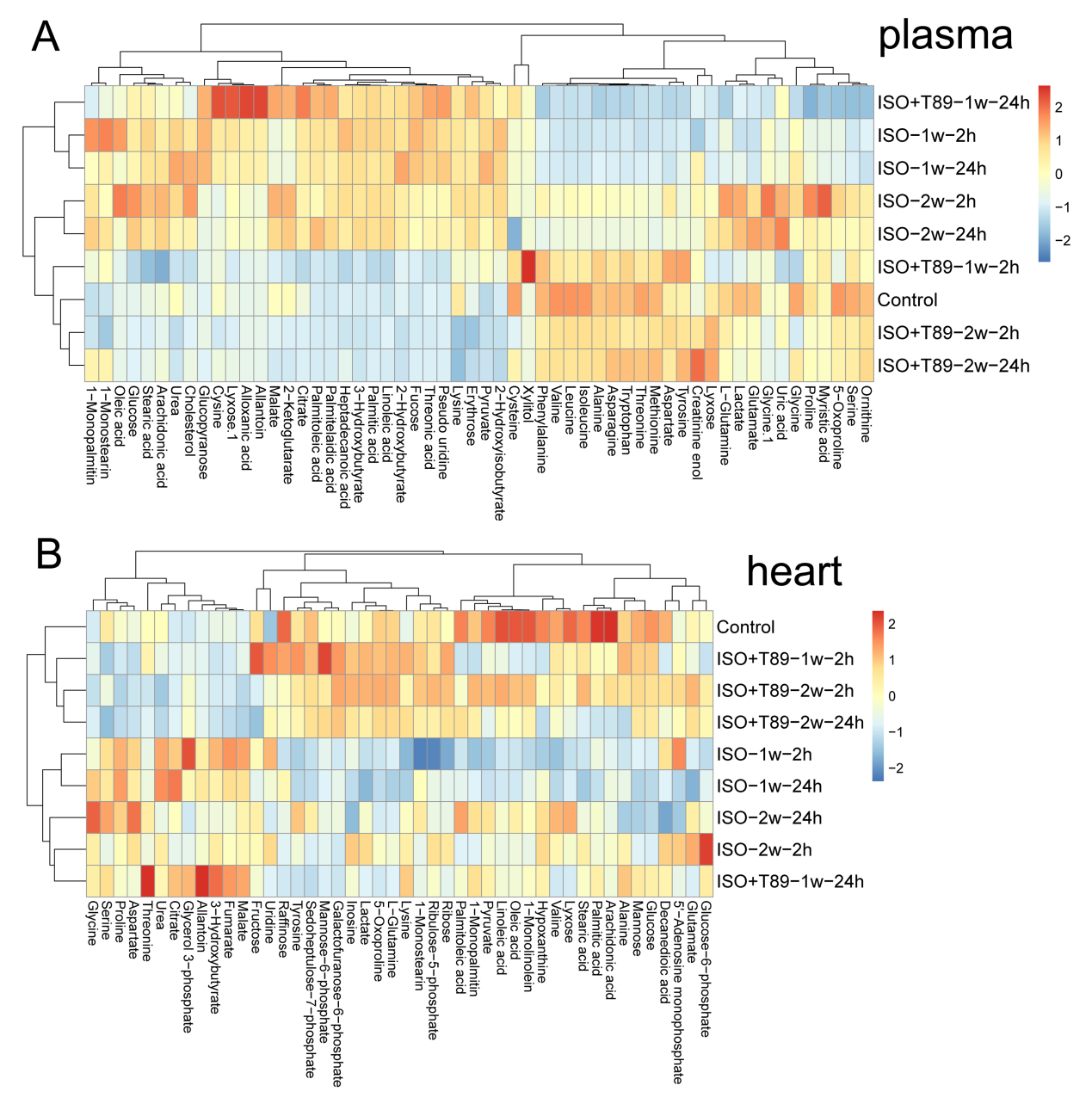


**Fig S5** Heatmap detailing the metabolome composition and energy substrates in the plasma (A) and heart tissue (B) from each group(n=6). The color key represents the calibrated contents of each metabolite. Deeper reds indicate higher amounts of the metabolites, while darker blues indicate lower concentrations of the metabolites. The dendrogram on the left shows the clustering of groups with similar metabolite compositions (T89, CDDP).


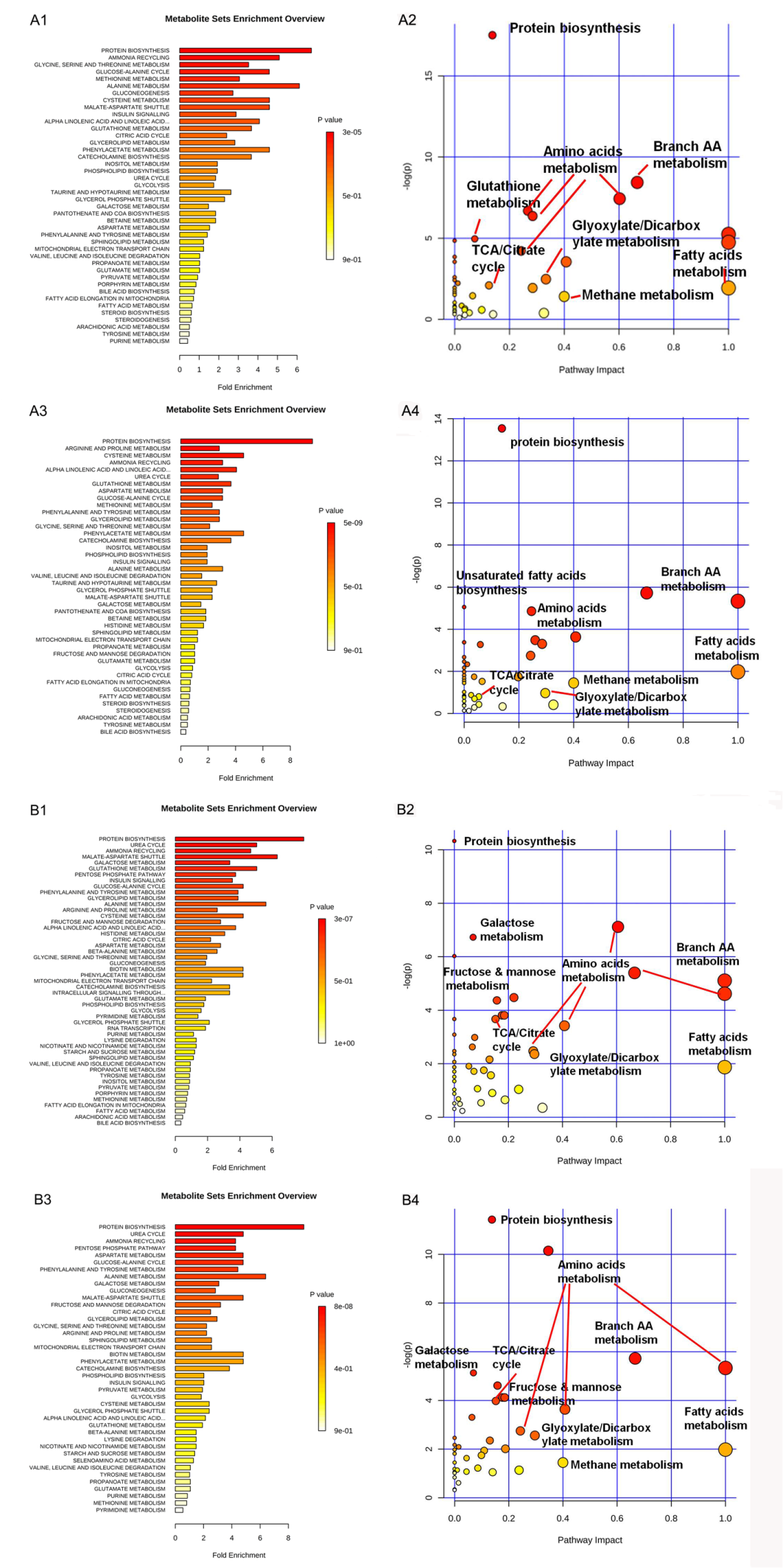


**Fig S6** Impact analysis of the metabolic pathways involved in the induction of ISO and the treatment with Dantonic (CDDP, T89) in myocardial ischemic rats based on the metabolomes of the plasma (A) and heart tissue (B).

A1 and A3: Enrichment analysis of metabolic sets induced by 1 or 2 weeks of ISO, respectively, relative to the normal control, based on the plasma metabolome;

A2 and A4: Impact analysis of metabolic sets induced by 1 or 2 weeks of ISO, respectively, relative to the normal control, based on the plasma metabolome;

B1 and B3: Enrichment analysis of metabolic sets induced by 1 or 2 weeks of ISO, respectively, relative to the normal control, based on the myocardial metabolome;

B2 and B4: Impact analysis of metabolic sets induced by 1 or 2 weeks of ISO, respectively, relative to the normal control, based on the myocardial metabolome.

**

**

**Fig S7** Boxplots of discriminant metabolites identified in the plasma (A) and heart tissue (B). Data are presented as the means ± SD(n=6). Statistical significance was evaluated using one-way ANOVA. ***P*< 0.01 vs control; **P*< 0.05 vs control; ##*P* < 0.01 vs model; #*P* < 0.05 vs model.

**
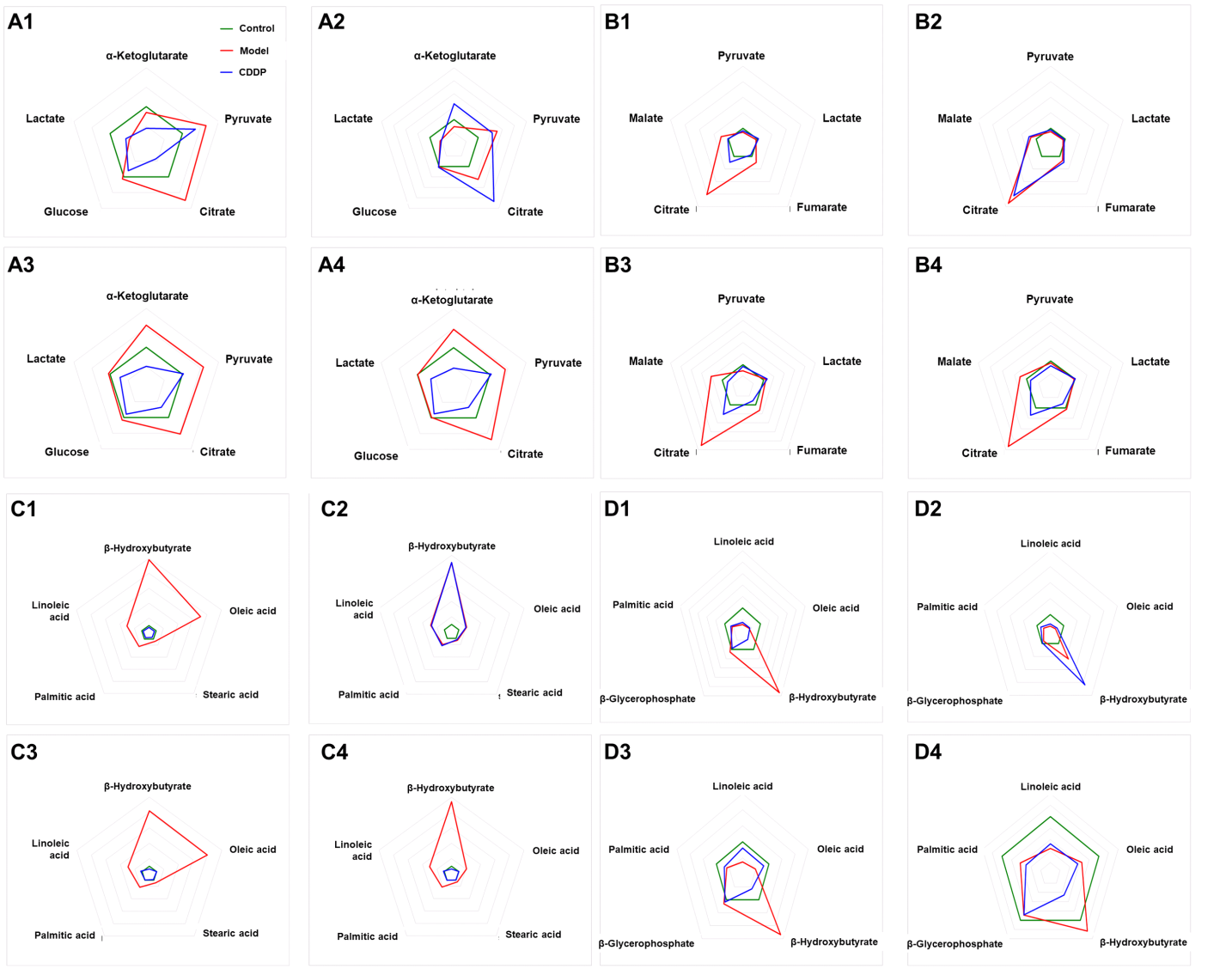
**

**Fig S8** A phase diagram of the key molecules in the plasma and heart tissue primarily involved in glycolysis, the TCA cycle and fatty acid metabolism. The phase diagram shows that ISO induced deviations in the TCA cycle and free fatty acid metabolism, especially in the pivotal molecules of citrate and 3-hydroxybutyrate, while CDDP restored metabolic homeostasis(n=6).

A1-A4: glycolysis and TCA intermediates in plasma; B1-B4: glycolysis and TCA intermediates in heart tissue; C1-C4: metabolites involved in fatty acid metabolism in plasma; D1-D4: metabolites involved in fatty acid metabolism in heart tissue. Odd numbers 1 and 3: 2 h after CDDP treatment. Even numbers 2 and 4: 24 h after CDDP treatment.

**
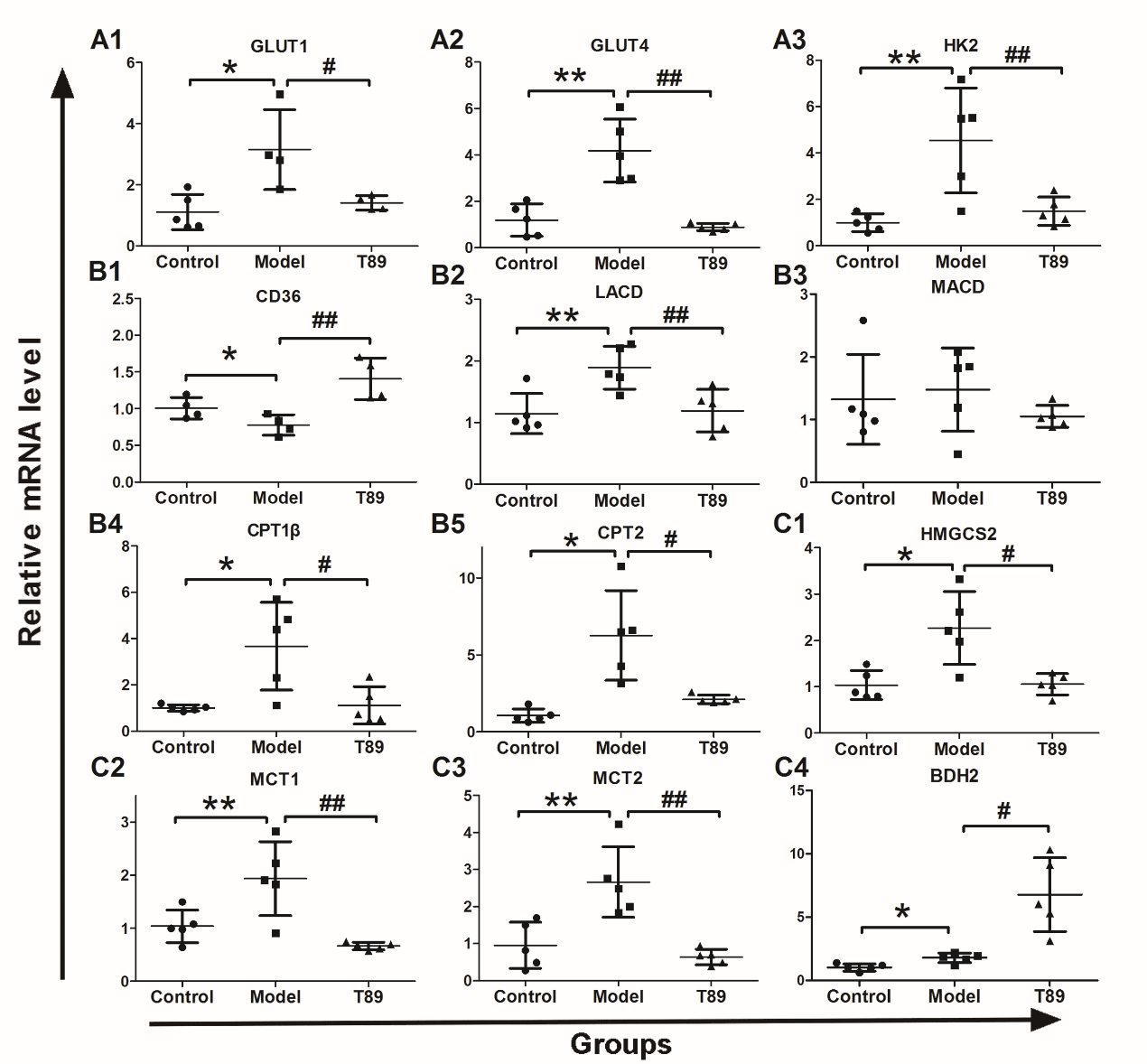
**

**Fig S9** Real-time quantitative PCR analysis of enzymes and transporters in the hearts and livers of rats induced by ISO or those treated with CDDP. Relative mRNA levels in each sample were normalized to those of β-actin. Data are presented as the means ± SD(n=5). Statistical significance was evaluated using one-way ANOVA. **P< 0.01 vs control; **P*< 0.05 vs control; ##*P*< 0.01 vs model; #*P*< 0.05 vs model. A, B: samples from rats’ heart. C: samples from the liver.
