# Supplementary material for: Compound danshen dripping pills normalize a reprogrammed metabolism of myocardial ischemia rats to interpret its time-dependent efficacy in clinic trials: a metabolomic study
Source: Metabolomics. 2019 Sep 20;15(10):128. doi: 10.1007/s11306-019-1577-3 (PMC6754357; doi:10.1007/s11306-019-1577-3)
Supplement: Supplementary file 2 — Supplementary Tables (Tables S1–S7) (DOCX 61 kb) [file 11306_2019_1577_MOESM2_ESM.docx]

**Compound danshen dripping pills normalizes a reprogrammed metabolism of myocardial ischemia rats to interpret its time-dependent efficacy in clinic trials: a metabolomic study**

**Supplementary Tables**

**Tab S1 The contents and limits of the primary components in CDDP**

| Components | Content assay  (mg/g) | Limits ragne  (mg/g) | Notes （Products 20130305） |
| --- | --- | --- | --- |
| Tanshenol | 10.74 | 9.33-12.59 |  |
| Ginsenoside Rg1 | 5.85 | 5.11-7.70 |  |
| Poly-phenolic acids | 23.04 | 18.85-28.26 | Including: Protocatechuic aldehyde, Salvianolic acid B, Salvianolic acid A, Rosmarinic acid |
| poly-saponins | 13.11 | 11.56-17.33 | Including: Notoginsenoside R1, Ginsenoside Re, Ginsenoside Rb1 |

**Tab S2 Primer sets for PCR assay of enzymes and transporters in rats’ heart and liver**

| Name | Forward Primer(5'-3') | Reverse Primer(5'-3') |
| --- | --- | --- |
| Rattus-Glut1 | GCTGTGGCTGGCTTCTCTAAC | CCGGAAGCGATCTCATCGAA |
| Rattus- Glut4 | GGTGCTATGGCTCCACTCTG | AGATGAGTGGGGGCGATTTC |
| Rattus-CPT1β | ACGTGTGCATGCCACCCCTTATG | AAGTTATCGAGTTCAGAAACG |
| Rattus-CPT2 | TTTGAGACTGGCGTTGGGAAG | GCCCTGGTAAGCTGGTCATT |
| Rattus-BDH2 | GGTGCATGTTTTTGGATTGCACT | ACTTTGGCTCCTTCTCTTGCGA |
| Rattus-CD36 | ATAACTGGATTCACTCTACAGTTTGC | GATCTGCAAGCACAGTATGAAATC |
| Rattus-MCT1 | GCTGCTTCTGTTGTTGCGAA | AAATCCAAAGACTCCCGCGT |
| Rattus-LACD | GCTGGGGTTACATGTGGGAA | GTACCACCGTAGATCGGCTG |
| Rattus-MACD | CTGGTGCTGTCGGGCTAG | AGGCTGCTCGCTGGTAAC |
| Rattus-HK2 | CTGGTGAGCCATCGTGGTTA | AAGCAGGCGATCATATGCGA |
| Rattus-MCT2 | AATCAAGCTGGCCCAAACCT | GGTGAGTGAGAGCAGCGTAA |
| Rattus-Hmgcs1 | TTTGGTGCCTGAAGGAGGAAC | GCATGGTGAAAGAGCTGTGTG |
| Rattus-β-actin | TCAGGTCATCACTATCGGCAAT | AAAGAAAGGGTGTAAAACGCA |

Glut1：Glucose transporter 1; Glut4：Glucose transporter 4; CPT1β：Carnitine palmitoyltransferase 1β; CPT2：Carnitine palmitoyltransferase 2; BDH2：3-hydroxybutyrate dehydrogenase 2; CD36：Fatty acid transport protein; MCT1：Monocarboxylate transporter 1; LACD：Long chain acyl-CoA dehydrogenase; MACD：Medium chain acyl-CoA dehydrogenase; HK2：Hexokinase 2; MCT2：Monocarboxylate transporter 2; Hmgcs1：Hydroxy-3-methylglutaryl-CoA.

**Tab S3 Histopathology severity scores for myocardial tissue**

| Group | No. | Description: myocardial structural disorder, myocardial cell necrosis, inflammatory cell infiltration, sub-endocardial hyperemia and edema, and local collagen fiber hyperplasia |
| --- | --- | --- |
| Normal control | 1 | - |
|  | 2 | - |
| ISO model 1week-2h | 1 | +++ |
|  | 2 | +++ |
| ISO model 1week-24h | 1 | ++ |
|  | 2 | +++ |
| ISO model 2week-2h | 1 | +++ |
|  | 2 | ++ |
| ISO model 2week-24h | 1 | ++ |
|  | 2 | +++ |
| ISO+T89 | 1 | ++ |
| 1week-2h | 2 | ++ |
| ISO+T89 | 1 | + |
| 1week-24h | 2 | ++ |
| ISO+T89 | 1 | + |
| 2week-2h | 2 | + |
| ISO+T89 | 1 | ± |
| 2week-24h | 2 | + |

“-”“±”“+”“++”“+++” indicates no lesion、minimal、mild、moderate and sever change respectively. The histopathology assessments were converted to numbers with minimal = 1, mild= 2, moderate = 3, severe = 4.

**Tab S4 The identified endogenous compounds in plasma**

| Identification | TMS | m/z | RT (min) |
| --- | --- | --- | --- |
| 3-Hydroxypyridine | 1TMS | 152 | 4.76 |
| Pyruvate | 1TMS | 174 | 4.93 |
| Lactate | 2TMS | 219 | 5.07 |
| 2-Hydroxyuisobutyrate | 2TMS | 205 | 5.12 |
| Glycolate | 2TMS | 205 | 5.23 |
| Alanine | 2TMS | 116 | 5.55 |
| Glycine | 3TMS | 204 | 5.71 |
| 3-Hydroxybutyrate | 2TMS | 233 | 6.13 |
| Monomethylphosphate | 2TMS | 241 | 6.31 |
| Urea1 | 2TMS | 261 | 6.38 |
| 2-Oxoisocaproate | MEOX1,TMS | 200 | 6.64 |
| 2-Ethylhydracrylic acid | 2TMS | 247 | 6.77 |
| Urea2 | 2TMS | 189 | 6.83 |
| 2-Aminoethanol | 2TMS | 174 | 7.12 |
| Leucine | 2TMS | 158 | 7.16 |
| Phosphate | 3TMS | 314 | 7.18 |
| Isoleucine | 2TMS | 158 | 7.34 |
| Proline | 2TMS | 142 | 7.38 |
| Glycine | 3TMS | 248 | 7.45 |
| Serine | 3TMS | 204 | 7.86 |
| Threonine | 3TMS | 291 | 8.07 |
| Aminovalerolactam | 2TMS | 243 | 8.56 |
| Aminomalonate | 3TMS | 320 | 8.65 |
| Aspartate | 3TMS | 232 | 8.68 |
| Malate | 3TMS | 233 | 8.75 |
| Erythritol | 4TMS | 307 | 8.92 |
| Methionine | 3TMS | 293 | 8.97 |
| 5-Oxoproline | 2TMS | 156 | 9.00 |
| Threonate | 4TMS | 292 | 9.16 |
| Cysteine | 3TMS | 220 | 9.21 |
| Creatinine | 3TMS | 329 | 9.25 |
| 2-Ketoglutarate | MEOX, 2TMS | 288 | 9.30 |
| Myristic acid | TMS | 216 | 9.37 |
| Ornithine | 3TMS | 348 | 9.56 |
| Glutamate | 3TMS | 246 | 9.58 |
| Phenylalanine | 2TMS | 218 | 9.67 |
| Asparagine | 3TMS | 188 | 9.91 |
| Lyxose | 4TMS | 307 | 10.0 |
| Xylitol | 5TMS | 307 | 10.19 |
| Glutamine1 | 4TMS | 156 | 10.28 |
| Fucose | 4TMS | 117 | 10.31 |
| Glycero-3-phosphate | 4TMS | 357 | 10.45 |
| Glutamine2 | 4TMS | 156 | 10.49 |
| Citrate | 4TMS | 273 | 10.77 |
| Inositol | 6TMS | 318 | 10.91 |
| Erythrose | 3TMS | 218 | 10.97 |
| Lyxose | 4TMS | 307 | 11.11 |
| Alloxanic acid | 4TMS | 331 | 11.16 |
| Glucose1 | MEOX2, 5TMS | 319 | 11.36 |
| Tyrosine | 3TMS | 218 | 11.39 |
| Maltose | MEOX2, TMS | 316 | 11.52 |
| Palmitoleic acid | 1TMS | 311 | 11.72 |
| Palmitelaidic Acid | 1TMS | 311 | 11.74 |
| Palmitic acid | 1TMS | 313 | 11.83 |
| Glucose2 | 5TMS | 319 | 11.88 |
| Allantoin | 3TMS | 259 | 12.03 |
| 3-Indole propanate | 4TMS | 333 | 12.13 |
| Urate | 4TMS | 441 | 12.21 |
| Myo-inositol | TMS | 318 | 12.23 |
| Heptadecanoic acid | 1TMS | 327 | 12.30 |
| Linoleic acid | 1TMS | 337 | 12.63 |
| Oleic acid | 1TMS | 339 | 12.67 |
| Stearic acid | 1TMS | 341 | 12.75 |
| Tryptophan | 3TMS | 291 | 12.78 |
| Cystine | 1TMS | 218 | 13.09 |
| Pseudo uridine | 5TMS | 424 | 13.26 |
| Arachidonic acid | 1TMS | 150 | 12.87 |
| Inositol-1-phosphate | 7TMS | 433 | 13.71 |
| Oleanitrile | 1TMS | 220 | 13.85 |
| 1-Monopalmitin | 2TMS | 371 | 14.20 |
| 1-Monostearin | 2TMS | 399 | 15.07 |
| 2-Tocopherol | 1TMS | 502 | 17.63 |
| Cholesterol | 1TMS | 458 | 17.90 |

MEOX，methoxymation; TMS, trimethylsilylation.

**Tab S5 The identified endogenous compounds in heart tissue**

| Identification | TMS | m/z | RT (min) |
| --- | --- | --- | --- |
| 3-Hydroxypyridine | 1TMS | 152 | 4.78 |
| Pyruvate | MEOX TMS | 174 | 4.95 |
| 1,3 Propanediol | 2TMS | 130 | 5.02 |
| Lactate | 2TMS | 191 | 5.09 |
| Alanine1 | 2TMS | 190 | 5.56 |
| Glycine | 2TMS | 204 | 5.73 |
| 3-Hydroxybutyrate | 2TMS | 233 | 6.15 |
| Monomethylphosphate | 2TMS | 241 | 6.33 |
| Urea1 | 3TMS | 261 | 6.40 |
| Valine | 2TMS | 144 | 6.69 |
| Urea2 | 3TMS | 189 | 6.85 |
| 2-Aminoethanol | 2TMS | 174 | 7.13 |
| Leucine | 2TMS | 158 | 7.17 |
| Phosphate2 | 3TMS | 314 | 7.35 |
| Proline1 | 2TMS | 142 | 7.39 |
| Glycine | 3TMS | 248 | 7.47 |
| Glycerate | 3TMS | 292 | 7.65 |
| Uracil | 2TMS | 256 | 7.70 |
| Fumarate | 2TMS | 245 | 7.71 |
| Serine | 3TMS | 218 | 7.87 |
| Alanine2 | 2TMS | 116 | 8.01 |
| Threonine | 3TMS | 218 | 8.08 |
| Decanedioic acid | 2TMS | 373 | 8.28 |
| Aspartate1 | 2TMS | 160 | 8.30 |
| Nicotinamide | TBS 1X | 179 | 8.70 |
| Malate | 3TMS | 233 | 8.77 |
| Maleic acid | 2TMS | 245 | 8.83 |
| Aspartate2 | 2TMS | 100 | 8.98 |
| Proline2 | 2TMS | 156 | 9.02 |
| 6-Azathymine | 2TMS | 256 | 9.14 |
| Glutamate | 3TMS | 348 | 9.59 |
| Creatinine | 3TMS | 329 | 9.63 |
| Phenylalanine | 2TMS | 218 | 9.68 |
| Pyrophosphate | 4TMS | 451 | 9.92 |
| Aminoethanesulfonic acid | 2TMS | 326 | 9.95 |
| Lyxose | 4TMS | 307 | 10.01 |
| Glycero-3-phosphate | 4TMS | 243 | 10.26 |
| Glutamine1 | 4TMS | 227 | 10.29 |
| Glycerol-2-phosphate | 4TMS | 299 | 10.46 |
| Glutamine2 | 3TMS | 156 | 10.50 |
| O-Phosphorylethanolamine | 4TMS | 299 | 10.59 |
| Hypoxanthine | 2TMS | 265 | 10.71 |
| Citrate | 4TMS | 273 | 10.78 |
| Phenylethanolamine | 3TMS | 174 | 10.94 |
| Histamine |  | 312 | 11.07 |
| Fructose | 5TMS | 307 | 11.17 |
| Mannose | 5TMS | 319 | 11.26 |
| Lysine | 4TMS | 156 | 11.30 |
| Glucose | 5TMS | 319 | 11.37 |
| Tyrosine | 3TMS | 218 | 11.40 |
| Mannitol | 6TMS | 319 | 11.44 |
| Sorbitol | 1TMS | 319 | 11.48 |
| Sebacic acid | 2TMS | 373 | 11.55 |
| Maltose | MEOX2 TMS | 361 | 11.62 |
| Pantothenic acid | 3TMS | 291 | 11.68 |
| Palmitoleic acid | 1TMS | 311 | 11.76 |
| 2,6-hydroxy-9H-Purine | 3TMS | 353 | 11.81 |
| Heptadecanoic acid | 1TMS | 313 | 11.84 |
| Palmitic acid | 4TMS | 313 | 11.88 |
| Pentanoic acid | 1TMS | 299 | 11.99 |
| Allantoin | 4TMS | 259 | 12.04 |
| Urate | 4TMS | 441 | 12.21 |
| Myo-inositol | 6TMS | 265 | 12.24 |
| Ribose | 1TMS | 315 | 12.30 |
| Ribulose 5-phosphate | 5TMS | 357 | 12.35 |
| Glucitol | 6TMS | 319 | 12.49 |
| Glucose-6-Phosphate | 6TMS | 387 | 12.55 |
| Linoleic acid | 1TMS | 337 | 12.64 |
| Oleic acid | 1TMS | 339 | 12.68 |
| Stearic acid | 1TMS | 341 | 12.76 |
| Galactofuranose-6-Phosphate1 | 6TMS | 387 | 13.18 |
| Mannose-6-Phosphate | 6TMS | 387 | 13.29 |
| Arachidonic acid1 | 1TMS | 117 | 13.34 |
| Mannose-6-Phosphate3 | 6TMS | 387 | 13.39 |
| Arachidonic acid | 1TMS | 117 | 13.45 |
| 11,14-Eicosadienoic acid | 1TMS | 365 | 13.50 |
| Arachidonic acid4 | 1TMS | 117 | 13.55 |
| Myo-Inositol-2-Phosphate | 7TMS | 318 | 13.72 |
| Uridine | 3TMS | 259 | 13.77 |
| 1-O-heptadecyl glycerol | 2TMS | 205 | 13.80 |
| Cis-Docosahexaenoic acid | 1TMS | 159 | 14.14 |
| Arachidonic acid5 | 1TMS | 117 | 14.20 |
| 1-Monopalmitin | 2TMS | 371 | 14.22 |
| Inosine | 4TMS | 245 | 14.26 |
| Sedoheptulose-7-Phosphate | 7TMS | 471 | 14.30 |
| Glucuronoic lactone | 3TMS | 230 | 14.53 |
| 1-Monolinolein | 2TMS | 395 | 14.99 |
| Galactofuranose-6-Phosphate | 6TMS | 387 | 15.06 |
| 1-Monostearin | 2TMS | 399 | 15.09 |
| Raffinose | TMS | 361 | 15.16 |
| Maltose2 | MEOX1 TMS | 361 | 15.37 |
| Adenosine-5-monophosphate | 5TMS | 315 | 17.14 |
| Tocopherol | 1TMS | 502 | 17.65 |
| Cholesterol | 1TMS | 368 | 17.91 |
| Maltose3 | MEOX1 TMS | 361 | 18.90 |

MEOX，methoxymation; TMS, trimethylsilylation.

**Tab S6 The perturbation of plasma metabolites in model rats and the modulation of CDDP**

| Metabolites | Fold change and statistical significance | | | | | | | |
| --- | --- | --- | --- | --- | --- | --- | --- | --- |
|  | I week | | | | II week | | | |
|  | Model/Control | Model/Control | Drug/Model | Drug/Model | Model/Control | Model/Control | Drug/Model | Drug/Model |
|  | (2h) | (24h) | (2h) | (24h) | (2h) | (24h) | (2h) | (24h) |
| Glucose | 1.55** | 1.45** | 0.46** | 1.01 | 2.10** | 1.83** | 0.43** | 0.51** |
| Fructose | 3.58** | 3.42** | 0.30** | 0.99 | 2.42** | 2.16** | 0.44** | 0.51** |
| Lyxose | 1.84 | 1.57* | 0.39* | 1.84 | 1.4 | 1.32 | 0.55** | 0.73* |
| Pyruvate | 1.65* | 1.78** | 0.82 | 0.89 | 1.58** | 1.43** | 0.65** | 0.73** |
| Lactate | 0.47** | 0.57** | 1.2 | 0.93 | 1.04 | 0.99 | 0.69* | 0.64** |
| Citrate | 1.75** | 1.62** | 0.24** | 1.65** | 1.52** | 1.69** | 0.44** | 0.39** |
| Aspartate | 0.38** | 0.36** | 3.80** | 0.68* | 1.03 | 0.67 | 1.18 | 1.56 |
| Malate | 0.74 | 0.77 | 0.66* | 2.42 | 1.82** | 1.58* | 0.31** | 0.28** |
| 2-keto-glutarate | 0.85 | 0.73 | 0.52** | 2.22 | 1.57* | 1.47* | 0.32** | 0.32** |
| Glutamine | 0.73* | 0.70** | 0.87 | 0.78 | 1.06 | 0.85 | 0.82 | 0.99 |
| Glutamate | 0.44** | 0.46** | 1.43** | 1.05 | 0.92 | 1.08 | 0.75 | 0.64** |
| Alanine | 0.35** | 0.40** | 2.78** | 0.58* | 0.67** | 0.53** | 1.36* | 1.75** |
| Glycine | 0.64 | 0.76 | 0.45 | 0.41* | 0.87 | 0.66 | 0.45** | 1.18 |
| Valine | 0.19 ** | 0.20** | 3.62** | 0.77 | 0.46** | 0.35** | 1.58** | 2.11** |
| Leucine | 0.19** | 0.20** | 3.49** | 0.84 | 0.54** | 0.39** | 1.43** | 1.83** |
| Isoleucine | 0.18** | 0.20** | 3.63** | 0.88 | 0.50** | 0.38** | 1.55** | 1.96** |
| 2-Hydroxybutyrate | 3.24* | 4.34** | 0.31* | 0.8 | 2.89** | 2.91** | 0.17** | 0.20** |
| Proline | 0.62* | 0.67* | 1.34* | 0.39** | 1.29 | 0.88 | 0.58* | 0.9 |
| Serine | 0.46 ** | 0.48** | 1.76** | 0.61* | 0.84 | 0.70* | 0.94 | 1.13 |
| Threonine | 0.32** | 0.37** | 2.36** | 0.84 | 0.57** | 0.50** | 1.35* | 1.78** |
| Methionine | 0.41** | 0.47** | 2.13** | 0.78 | 0.57** | 0.50** | 1.52** | 1.95** |
| 5-Oxoproline | 0.67* | 0.66** | 1.01 | 0.79 | 0.91 | 0.78 | 0.92 | 1.05 |
| Cysteine | 0.69* | 0.69* | 1.30* | 1.25* | 0.70* | 0.41** | 0.85 | 1.94** |
| Ornithine | 0.48** | 0.42** | 1.47* | 0.69 | 0.82 | 0.76 | 1.1 | 1.23 |
| Tryptophan | 0.37** | 0.38** | 2.52** | 0.70** | 0.63** | 0.53** | 1.28 | 1.89** |
| Phenylalanine | 0.61** | 0.59** | 1.61** | 0.81 | 0.81 | 0.64** | 1.05 | 1.40** |
| Asparagine | 0.31** | 0.35** | 2.68** | 0.59** | 0.61* | 0.48** | 1.43* | 2.08** |
| Lysine | 1.02 | 1.01 | 0.83 | 1.02 | 1.05 | 0.95 | 0.56* | 0.60* |
| Tyrosine | 0.56** | 0.64* | 2.55** | 0.67** | 0.70* | 0.74* | 1.62* | 1.80** |
| Cystine | 1.25* | 1.19* | 0.71** | 1.58** | 0.81** | 0.89 | 0.85 | 0.98 |
| Palmitoleic acid | 8.76 | 8.47* | 0.1 | 1.36 | 6.1 | 11.34** | 0.17 | 0.10** |
| Palmitelaidic acid | 7.77** | 6.59** | 0.12** | 1.57* | 7.35** | 8.54** | 0.16** | 0.16** |
| Palmitic acid | 2.22* | 2.00** | 0.34** | 1.09 | 2.14** | 2.12** | 0.45** | 0.48** |
| Heptadecanoic acid | 1.94 | 1.75** | 0.42* | 0.95 | 1.77** | 1.62** | 0.52** | 0.67** |
| Linoleic acid | 3.03** | 2.88** | 0.26** | 0.97 | 2.94** | 3.02** | 0.41** | 0.37** |
| Oleic acid | 7.12* | 2.07** | 0.11* | 0.96 | 7.98 | 2.07** | 0.13 | 0.49** |
| Stearic acid | 1.37 | 1.31** | 0.52* | 0.92 | 1.42** | 1.29** | 0.64** | 0.76** |
| Arachidonic acid | 1.16 | 1.22* | 0.60** | 0.85 | 1.33** | 1.26* | 0.71** | 0.79** |
| 3-Hydroxybutyrate | 9.82** | 9.05** | 0.08** | 1 | 8.22 ** | 9.42** | 0.08** | 0.07** |
| Pseudo uridine | 2.82** | 3.12** | 0.30** | 1.15 | 2.13** | 2.27** | 0.47** | 0.45** |
| Allantoin | 4.11** | 3.91** | 0.20** | 2.64** | 1.70** | 2.39** | 0.53** | 0.37** |
| Urea | 1.23 | 1.47** | 0.50* | 0.60** | 1.26 | 0.94 | 0.45 | 0.77 |

Statistical significance was evaluated using one-way analysis of variance. *:p<0.05; **:p<0.01.

**Tab S7 The perturbation of myocardial metabolites in model rats and the modulation of CDDP**

| Metabolites | Fold change and statistical significance (Student’s T-test, two tailed, unpaired) | | | | | | | |
| --- | --- | --- | --- | --- | --- | --- | --- | --- |
|  | I week | | | | II week | | | |
|  | Model/Control | Model/Control | Drug/Model | Drug/Model | Model/Control | Model/Control | Drug/Model | Drug/Model |
|  | (2h) | (24h) | (2h) | (24h) | (2h) | (24h) | (2h) | (24h) |
| Glucose | 0.46** | 0.39** | 1.90** | 1.91** | 0.46** | 0.38** | 1.79** | 2.03** |
| Fructose | 0.89 | 0.75 | 1.44 | 1.09 | 0.92 | 0.94 | 0.77 | 0.61 |
| Mannose | 0.55** | 0.45** | 1.70* | 1.86** | 0.56** | 0.44** | 1.63** | 1.91** |
| Pyruvic acid | 0.56** | 0.62* | 1.27 | 1.30** | 0.73 | 0.86 | 1.27 | 0.78 |
| Lyxose | 0.27** | 0.33** | 2.21** | 0.97 | 0.48** | 0.85 | 1.16 | 0.49** |
| Raffinose | 0.09* | 0.46 | 9.47** | 0.27 | 0.18* | 0.24* | 2.19 | 1.56 |
| Galactofuranose-6-phosphate | 0.11* | 0.43 | 20.33** | 0.14 | 0.12* | 0.57 | 17.71* | 3.29 |
| Glucose-6-phosphate | 0.64 | 0.79 | 1.05 | 1.48 | 1.78* | 0.94 | 0.65* | 1.15 |
| Mannose-6-phosphate | 0.28 | 0.11 | 12.18** | 0.55 | 0.26 | 0.44 | 5.83* | 4.23 |
| Ribulose-5-phosphate | 0.50** | 0.79 | 1.98** | 1.09 | 1.02 | 0.8 | 1.07 | 1.2 |
| Sedoheptulose-7-Phosphate | 0.12* | 0.11* | 8.07** | 1.11 | 0.23* | 0.73 | 3.76** | 1.19 |
| Ribose | 0.59** | 0.85 | 2.16** | 1 | 1.12 | 0.87 | 1.1 | 1.33* |
| Fumarate | 2.20** | 1.76** | 0.36** | 1.23 | 1.3 | 1.13 | 0.59* | 0.57** |
| Malate | 2.27** | 1.86* | 0.48** | 1.2 | 1.54 | 1.6 | 0.48* | 0.43** |
| Citrate | 16.29** | 22.38* | 0.13** | 0.76 | 3.26 | 8.08 | 0.47 | 0.22 |
| Glutamate | 0.82 | 0.67** | 1.08 | 1.23 | 1.18 | 1.04 | 0.99 | 1.05 |
| Glutamine | 0.46** | 0.32** | 2.33** | 1.52 | 0.62** | 0.47** | 1.76** | 2.08** |
| Proline | 1.41* | 1.47* | 0.55** | 0.69* | 1.07 | 1.32 | 0.64* | 0.54** |
| Aspartic acid | 1.71 | 1.47 | 0.36* | 0.73 | 1.60* | 2.22* | 0.44** | 0.31* |
| Glycine | 1.2 | 1.61* | 0.9 | 0.88 | 1.41 | 1.93** | 0.67* | 0.52** |
| Lysine | 0.59* | 0.72 | 2.84** | 2.23** | 1.37* | 1.2 | 0.94 | 1.27 |
| Tyrosine | 0.46** | 0.50** | 2.76** | 1.08 | 0.73* | 1.12 | 1.34 | 0.79 |
| Leucine | 0.27** | 0.36** | 2.77** | 1.1 | 0.67* | 1.07 | 1.16 | 0.56* |
| Alanine | 0.76 | 0.8 | 1.41* | 0.96 | 0.70* | 1.01 | 1.22 | 1.08 |
| Threonine | 0.9 | 0.92 | 1.15 | 1.50** | 0.93 | 1.06 | 0.9 | 0.82 |
| 5-Oxoproline | 0.56** | 0.52** | 1.85** | 1.09 | 0.76* | 0.58** | 1.42** | 1.69** |
| Phenylalanine | 0.48** | 0.51** | 1.93** | 1.12 | 0.79 | 1.07 | 1.09 | 0.62* |
| Linoleic acid | 0.28** | 0.28** | 1.35* | 1.51* | 0.37** | 0.45** | 2.16** | 1.18 |
| Oleic acid | 0.66** | 0.56** | 1.05 | 1.31** | 0.70* | 0.72* | 1.46** | 1.04 |
| Palmitic acid | 0.58** | 0.49** | 1.13 | 1.44 | 0.60* | 0.62 | 1.16 | 0.81 |
| Palmitoleic acid | 0.37** | 0.44** | 0.98 | 1.24 | 0.41** | 0.93 | 1.35 | 0.84 |
| Stearic acid | 0.84 | 0.81* | 1.12 | 1.1 | 0.93 | 0.87 | 1.05 | 0.97 |
| Arachidonic acid | 0.74** | 0.76** | 1.09 | 1.01 | 0.78** | 0.75** | 1.1 | 0.95 |
| 3-Hydroxybutyrate | 3.33** | 2.19** | 0.14** | 1.91 | 2.34* | 1.23 | 0.25** | 0.37** |
| 1-monopalmitin | 0.65** | 0.75 | 1.09 | 1.28 | 0.75* | 0.96 | 1.35* | 0.94 |
| 1-monosteatin | 0.81* | 0.97 | 1.29** | 0.96 | 0.92 | 1 | 1.13* | 0.99 |
| 1-monolinolein | 0.34** | 0.46** | 1.16 | 1.24 | 0.47** | 0.58* | 1.73* | 1.09 |
| Glycerol 3-phosphate | 3.58** | 1.97* | 0.36** | 1.43 | 1.30* | 1.01 | 1.24 | 1.68** |
| Uridine | 2.56** | 1.68* | 1.08 | 0.84 | 2.35** | 1.34 | 0.73 | 1.46 |
| AMP | 1.5 | 0.77 | 0.74 | 1.22 | 1.34 | 0.73 | 0.97 | 1.53** |
| Inosine | 0.97 | 0.85 | 1.29** | 1.2 | 1.25* | 0.77* | 1.04 | 1.54** |
| Uracil | 0.54** | 0.80* | 1.11 | 0.97 | 0.91 | 1.30* | 0.79 | 0.39** |
| Hypoxanthine | 0.47** | 0.67** | 1.15 | 1.14 | 0.86 | 0.85 | 0.85 | 0.60** |
| Allantoin | 3.03** | 3.31** | 0.34** | 1.98** | 1.93** | 1.61** | 0.56** | 0.63** |
| Statistical significance was evaluated using an unpaired Student’s T-test with two tails. *:p<0.05; **:p<0.01. | | | | | | | | |
