# Supplementary material for: Compound danshen dripping pills normalize a reprogrammed metabolism of myocardial ischemia rats to interpret its time-dependent efficacy in clinic trials: a metabolomic study
Source: Metabolomics. 2019 Sep 20;15(10):128. doi: 10.1007/s11306-019-1577-3 (PMC6754357; doi:10.1007/s11306-019-1577-3)
Supplement: Supplementary file 3 — Supplementary methods (Method S1–S3) (DOCX 20 kb) [file 11306_2019_1577_MOESM3_ESM.docx]

**Compound danshen dripping pills normalizes a reprogrammed metabolism of myocardial ischemia rats to interpret its time-dependent efficacy in clinic trials: a metabolomic study**

**Supplementary Methods**

**Method S1**

**Agents.**

The stable-isotope-labeled internal standard compound (IS) myristic-1,2-^13^C_2_ acid (99 atom%^13^C), methoxyamine hydrochloride (purity 98%), and pyridine (≥99.8% GC) were provided by Sigma-Aldrich (St. Louis, USA). N-Methyl-N-trimethylsilyltrifluoroacetamide and 1% trimethylchlorosilane were purchased from Thermo Scientific (Bellefonte, USA). High-performance liquid chromatography grade methanol and n-heptane were obtained from Merck (Darmstadt, Germany). Purified water was produced using a Milli-Qsystem (Bedford, USA). All other reagents were of analytical grade.

**Method S2**

**Animal experiments and sample preparation.**

The animals were housed in polypropylene cages (each cage housed a maximum of 3 animals) for one week to enable their adaptation to the environment at ambient temperature (25±5°C) and at 45±5% relative humidity with a 12-hour light/dark cycle (lights on from 6:00 a.m. to 6:00 p.m.), as well as a standard diet and unrestricted access to water. The animals were fasted for 12 h before the experiment and for 4 h during the sample collection; however, free access to water was allowed at all times.

The animals were randomly divided into three groups: 1) Control group (Z); 2) ISO model group (ISO); and 3) CDDP treatment group (CDDP+ISO) (Supporting information, **Fig S2)**. The ISO group and CDDP treatment group each contained two subgroups: a 1-week treatment group and a 2-week treatment group. To induce myocardial ischemia, the rats in the model group were pretreated with ISO (15 mg/kg) subcutaneously for 3 consecutive days. The rats were then divided into two subgroups, with one group receiving continuous injections of ISO for 1 week every other day, and the other group received injections for 2 weeks. The CDDP treatment group was paired with the ISO group; however, in addition to the induction by ISO, these rats were also given CDDP (167 mg/kg BW) dissolved in distilled water (10 ml/kg) via daily intragastric administration. According to the parallel control principal, the normal control and ISO control rats were given distilled water or saline in the same way. At the end of the experiment, the rats were sacrificed  after anesthesia with 6% [chloral hydrate](http://www.baidu.com/link?url=mTQ8eO6KJ6gfOYEXV0UbW7UQ5ZeE8NdhyNcyNLCdpiQwbTauSDT6zUXvUQztBd9wN38LobrqlLx5rwlJbsZYFqNr8xBFMKGgxv3XAfAScNsqXadYWKQQIrMLQt1anDwH) solution ( 5 ml /kg, i.p.) 2 h and 24 h after CDDP administration. Blood samples (n=8) were collected in heparinized tubes via the retro-orbital plexus and centrifuged at 4000 rpm for 10 min at 4°C. The plasma was separated and stored at −70°C for biochemical analyses and the metabolomics study. Additionally, the heart and liver tissues (n=11, 2 h after CDDP administration) were quickly removed, washed with ice-cold saline, blotted dry with filter paper and stored at −70°C for later analyses. The other samples (n=3) were placed in 10% formalin solution for the pathological analyses. Myocardial tissues from each group were fixed in a 10% formalin solution for 48 h at room temperature. The tissues were dehydrated by sequential washes with 70%, 80%, 90% and 100% ethanol, embedded in paraffin wax (Leica EG1150, Germany) and cut into 3-5 μm slices (Leica RM 2235, Germany). One transversal section from the base area of the left ventricle was rehydrated and stained with hematoxylin and eosin (H&E) and examined via light microscopy (Nikon 80i, Japan) at 100 ×magnification by an experienced observer who was blinded to the groups.

**Method S3**

**Reverse transcription and real-time polymerase chain reaction analyses.**

The total RNA was isolated from the rat livers and hearts 2 weeks after the experiment using Trizol reagent in accordance with the manufacturer’s instructions. The RNA concentration was quantified using ultraviolet spectrophotometry. Gene expression levels were determined using the SYBR Green-based real-time PCR technique (RT-qPCR; Bio-Rad). The diluted mRNA (0.5 mg/ml) was reverse-transcribed using the Prime Script RT Reagent Kit Perfect Real Time (Takara Biomedicals). The primer sets used in this study are listed in supplementary Tab S1. The PCR conditions were as follows: 95°C for 15 s, 60°C for 30 s followed by 72°C for 30 s (39 cycles) and 65°C~95°C for 0.5°C/5 s. All the samples were quantified using the comparative Ct method for the relative quantification of gene expression, normalized to β-actin.
